# Reproducibility of an 85-item Child Food Frequency Questionnaire (85-item Child-FFQ) and diet quality scores among Polish children: The cross-sectional PITNUTS 2024 study

**DOI:** 10.1186/s12887-026-07222-6

**Published:** 2026-07-08

**Authors:** Michał Sawicki, Joanna Kowalkowska, Halina Weker, Lidia Wadolowska

**Affiliations:** 1 Nutricia Foundation, 8 Bobrowiecka Street, Warsaw, 00-728 Poland; 2 Department of Human Nutrition, Faculty of Food Science, University of Warmia and Mazury in Olsztyn, 45F Sloneczna Street, Olsztyn, 10-718 Poland; 3 Department of Nutrition, Institute of Mother and Child, 17a Kasprzaka Street, Warsaw, 01-211 Poland

**Keywords:** Food frequency questionnaire, Dietary assessment, Nutrition, Diet quality, Dietary pattern, Ultra-processed food, Reliability, Reproducibility, Toddlers, Preschoolers

## Abstract

**Background:**

Early childhood nutrition is crucial for long-term health; however, assessing dietary intake in young children is challenging due to the reliance on parent/caregiver reporting and the need for culturally relevant tools. The aim of this study (the Polish Infant and Toddler Nutrition Study, PITNUTS 2024) was to address this gap by developing and evaluating the reproducibility of a new 85-item Child Food Frequency Questionnaire (85-item Child-FFQ) and associated diet quality scores for Polish children aged 13–72 months.

**Methods:**

A cross-sectional, nationally representative sample of 700 Polish children from two age groups (13–36 and 37–72 months) was recruited, and 100 children were randomly selected from each age group for the reproducibility study. The 85-item Child-FFQ was administered twice to the same parent as a proxy reporter. Three diet quality scores were developed: the children’s pro-Healthy Diet Score, the children’s non-Healthy Diet Score, the children’s Ultra-Processed Food Score. Statistical analyses included comparisons of mean values for food consumption frequency and diet quality scores, cross-classification, kappa statistic, intraclass correlation coefficient (ICC), Spearman’s correlation, and Bland-Altman plots.

**Results:**

The 85-item Child-FFQ showed good test–retest reproducibility. Classification agreement for food consumption frequency ranged from 74% to 97% for children aged 13–36 months, and 77% to 95% for children aged 37–72 months. The kappa statistic indicated moderate to excellent agreement between test and retest of the FFQ. Diet quality scores demonstrated high reproducibility, with classification agreement ranging from 77% to 92%, the kappa statistic ranging from 0.62 to 0.86, and good to excellent correlation coefficients (ICC: 0.886–0.950, Spearman’s r: 0.889–0.947). The Bland-Altman analysis revealed a small negative shift between test and retest of the diet quality scores and moderately wide limits of agreement.

**Conclusions:**

The newly developed 85-item Child-FFQ is a reliable tool for assessing habitual food consumption frequency and diet quality in Polish children aged 13–72 months. This tool has the potential to help monitor food consumption and diet quality variations over time, though this capacity needs to be validated through future longitudinal studies.

**Trial registration:**

This trial was registered at www.clinicaltrials.gov (NCT06417151, 16th May 2024).

**Supplementary Information:**

The online version contains supplementary material available at https://doi.org/10.1186/s12887-026-07222-6.

## Background

Dietary practices during the first three years of life are critical due to the high prevalence of inadequate nutrient intake during this period [1], the tendency for early-established dietary patterns to persist throughout childhood [2], and the association between early-life diet and later health outcomes [3]. Dietary assessment in young children poses unique challenges. Children under 6 years of age cannot reliably self-report their intake. Thus, dietary data must be obtained via a parent or caregiver, often involving other people engaged in daycare. Food availability and eating habits vary by region and demographic. Assessment of habitual food consumption over a certain period requires considering country- or culture-specific food items and between-person variance in the consumption of target foods and dishes [4]. Food frequency questionnaires (FFQ) are used to assess usual diet due to their ease of administration and low cost, which is especially important in large-scale studies. They typically list a fixed set of foods with response options for consumption frequency.

Every FFQ is required to be validated in the target population, since systematic and random bias may affect the quality of a tool [5]. The number and type of validated tools available to assess the food consumption of the youngest children are limited [6–9]. In many cases, dietary questionnaires for children have been validated among school-age children or adolescents [10], or grouped toddlers with older children [11, 12]. It may potentially overestimates the validity and reliability for the toddler subgroup. In the early childhood group, children’s diets differ significantly, evolving into family-specific meals, and, consequently, should be validated in specific subgroups [13]. Several studies have addressed this gap, underscoring that an appropriately designed FFQ can capture young children’s diets with reasonable accuracy and reliability [7, 14–16].

In Poland, to address the need for a systematic assessment of children’s diets, the Polish Infants and Toddlers Nutritional Study (PITNUTS) was launched. In the PITNUTS 2016 study, detailed dietary data were collected on children aged 5–36 months across Poland [17]. Based on that experience, in the current PITNUTS 2024 study [18], the age range of children has been expanded to include children aged 5 to 72 months, and the development of a new FFQ suitable for this broader age span was necessary. The 85-item Child Food Frequency Questionnaire (85-item Child-FFQ) was developed by pediatric nutrition experts and modelled on experience with the previous PITNUTS 2016 questionnaire, with updates to include food items relevant to preschool-age children and contemporary dietary patterns. To the best of our knowledge, there are no qualitative FFQs available in Poland to assess food consumption in children under 6 years of age. Moreover, in order to holistically assess the quality of children’s diet, three diet quality scores derived from the data collected with the 85-item Child-FFQ were developed. This includes two opposing diet quality scores evaluating the pro-healthy and unhealthy characteristics of the children’s diet as well as the third score developed to assess frequency of consumption of ultra-processed food (UPF), which constitutes a large and growing share of young children’s diets, raising concern about their impact on nutrition and health outcomes [19, 20].

This study aimed to evaluate the test–retest reproducibility of the newly developed 85-item Child-FFQ and associated three diet quality scores among Polish children aged 13–72 months.

## Methods

### Study design and participants

The assessment of test-retest reproducibility of the 85-item Child-FFQ among Polish children was part of the PITNUTS 2024 study, a cross-sectional study that included a representative sample of 1,000 children aged 5–72 months [18]. In the present study, test-retest reproducibility of the 85-item Child-FFQ and three diet quality scores developed for children aged 13–72 months was evaluated among 200 children randomly selected from a representative sample of the PITNUTS 2024 study (*n* = 100 from each 13–36 months and 37–72 months age groups). The study questionnaires were completed by parents as proxy reporters. The test-retest reproducibility of the 85-item Child-FFQ was assessed by administering the questionnaire twice to the same parent, with a 14-day interval between both administrations. The dietary interviews were conducted by the same interviewer in test and retest of the questionnaire to reduce variability in data collection.

### 85-item child food frequency questionnaire

The 85-item Child-FFQ is a qualitative food frequency questionnaire (FFQ) developed for the PITNUTS 2024 study by Kowalkowska, Sawicki, and Weker in 2024, based on the questionnaire used in the previous Polish Infants and Toddler Nutritional Study carried out in 2016 (PITNUTS 2016) among children aged 5–36 months [10]. The 85-item Child-FFQ is designed to assess habitual food consumption over the past month. This FFQ includes a list of 85 food items and contains a set of six food frequency categories to choose from, which for further analysis have been converted into daily frequency of food consumption using coefficients ranging from 0 to 2 times/day: (1) never or almost never (0 times/day), (2) less than once a week (0.06), (3) once a week (0.14), (4) 2–6 times a week (0.57), (5) once a day (1.00), (6) several times a day (2.00). These coefficients have been established based on the literature and our own experiences in developing and interpreting data from FFQs [10, 21, 22].

### Diet quality scores

Three diet quality scores were developed for children aged 13–72 months (1–6 years) to comprehensively assess their diet quality:

- Children’s pro-Healthy Diet Score (child-pHDS) – consists of 7 food groups: vegetables, pulses/nuts, fruits, cereal products, unsweetened dairy products, meat/fish/eggs, and recommended fats, and includes a total of 25 food items from the 85-item Child-FFQ (Supplementary Table S1),
- Children’s non-Healthy Diet Score (child-nHDS) – consists of 10 food groups: sweetened breakfast cereals, fried flour-based dishes, fast food, sweetened dairy products, processed meat, food concentrates/salty seasonings, sweet condiments, sweets, salty snacks, sweetened beverages, and includes a total of 28 food items from the 85-item Child-FFQ (Supplementary Table S2),
- Children’s Ultra-Processed Food Score (child-UPFs) – consists of 10 food groups: ready-to-eat breakfast cereals, fast food, sweetened dairy products, processed meat, savoury condiments, food concentrates/salty seasonings, sweet condiments, sweets, salty snacks, beverages, and includes a total of 27 food items from the 85-item Child-FFQ (Supplementary Table S3).

Based on an extensive literature review [19, 23–26] and the research team’s established expertise in FFQ methodology and data processing [10, 21, 22, 27], these newly developed diet quality scores were tailored specifically to the data obtained from the 85-item Child-FFQ established within the PITNUTS 2024 study. The diet quality scores (child-pHDS, child-nHDS) were developed mainly based on the official position statements on pediatric nutrition in Poland [23, 24]. For the child-UPFs, food items were categorized based on the NOVA system [25, 26], with the exclusion of products that could be either homemade or commercially manufactured (e.g., cakes, cookies).

The consumption frequency of each food group was calculated by summing the daily consumption frequency (times/day) of the included food items in each of the food groups. Then, a median consumption frequency was calculated for each food group based on the results of the PITNUTS 2024 project, separately for the two age groups of children: 13–36 months and 37–72 months (Supplementary Table S4). For each respondent, the daily consumption frequency of a given food group above the median was scored 1 point. The score of each of the three diet quality scores was calculated as the sum of the points obtained by a given respondent. Therefore, the child-pHDS ranged from 0 to 7 points, and the child-nHDS ranged from 0 to 10 points. Next, the child-pHDS was categorised into three categories reflecting the level of pro-healthy characteristics of the diet: low (0–2 points), moderate (3–5 points), and high (6–7 points). The child-nHDS was categorised into three categories reflecting the level of unhealthy characteristics of the diet: low (0–3 points), moderate (4–7 points), and high (8–10 points). The child-UPFs was categorised into three categories reflecting the level of ultra-processed food consumption frequency: low (0–3 points), moderate (4–7 points), and high (8–10 points), not considering the potential adverse or beneficial effects of these foods.

### Statistical analysis

Participant characteristics were expressed as mean values and standard deviations (SD) for continuous variables and percentage distribution for categorical variables. Test-retest reproducibility of the 85-item Child-FFQ and diet quality scores was evaluated in three age groups of children using the following analyses: (i) comparison of mean values of the diet quality scores and the food consumption frequency using the Wilcoxon signed-rank test (for two dependent samples), (ii) cross-classification analysis, (iii) kappa statistics, (iv) the Spearman’s correlation coefficient (r) and intraclass correlation coefficient (ICC) with 95% confidence interval (CI), and (v) the Bland-Altman plots for the diet quality scores.

In the cross-classification analysis, the percentage of children classified into the same category of diet quality scores or food consumption frequency above 50% indicated an acceptable level of agreement [28]. The agreement assessed with the kappa statistic was interpreted as follows: slight (0–0.20), fair (0.21–0.40), moderate (0.41–0.60), good (0.61–0.80), and excellent (0.81–1.00) [29]. Generally, a kappa statistic above 0.40 is considered acceptable [28]. The correlation strength was interpreted as follows: weak (0–0.29), moderate (0.30–0.49), good (0.50–0.69), and very good (0.70–1.00) [30]. The reliability assessed with the ICC was interpreted as follows: poor (0–0.49), moderate (0.50–0.74), good (0.75–0.89), and excellent (0.90–1.00) [31]. The Bland–Altman plots were used for diet quality scores to assess an agreement between test and retest in both age groups of children. Mean difference between test and retest and 95% limits of agreement (LOA) were calculated [32]. The internal reliability of the diet quality scores was evaluated using Cronbach’s alpha (Supplementary Tables S5, S6 and S7), with values ranging from 0.70 to 0.90 considered recommended [33].

The statistical analysis was conducted using STATISTICA 13 (TIBCO Software Inc., Cracow, Poland) and PS IMAGO PRO 10.0, including IBM SPSS Statistics 29 (Predictive Solutions, Cracow, Poland). P-values < 0.05 were considered statistically significant.

## Results

### Participant characteristics

The characteristics of the three age groups of children are presented in Table 1. Girls comprised 48.0% of children aged 13–36 months, and 57.0% of children aged 37–72 months. Rural areas were inhabited by 29.0% and 31.0% of children aged 13–36 and 37–72 months, respectively. On the other hand, in cities with ≥ 100,000 inhabitants lived 26.0% and 24.0% of children from each age group, respectively. Most parents/legal guardians of children declared a good economic situation in their household (67.0% and 61.0% of the respondents, respectively). Mothers with higher education had 49.0 and 37.0% of children aged 13–36 and 37–72 months, respectively, while fathers with higher education had 33.3% and 24.2% of children from each age group, respectively. Most of the children’s parents declared good nutrition knowledge (69.0% and 77.0% of the respondents, respectively). The child-pHDS was low in 48.0% of children aged 13–36 months and 45.0% of children aged 37–72 months, while high in 13.0% and 18.0% of children, respectively. The child-nHDS was low in 51.0% of children aged 13–36 months and 47.0% of children aged 37–72 months, while high in 11.0% and 17.0% of children, respectively. The child-UPFs was low in 58.0% of children aged 13–36 months and 45.0% of children aged 37–72 months, while high in 15.0% and 18.0% of children, respectively. Considering children’s physical activity (PA) level compared to peers, lower PA had 0%, and 5.0% of children aged 13–36 and 37–72 months, respectively, while higher PA had 16.0% and 20.0% of children from each age group, respectively. Based on BMI-for-age z-scores, normal weight was observed in 78.9% and 80.8% of children aged 13–36 and 37–72 months, respectively.

**Table 1:** Participant characteristics by children’s age groups

Variables	Children aged 13–36 months	Children aged 37–72 months
	mean (SD) or %	mean (SD) or %
Sample size	100	100
Sex		
boys	52.0	43.0
girls	48.0	57.0
Age (months)^1^	19.6 (6.2)	50.2 (9.2)
Type of municipality		
rural	29.0	31.0
urban-rural	23.0	24.0
urban ≤ 100,000 inhabitants	22.0	21.0
urban > 100,000 inhabitants	26.0	24.0
Economic situation of the household		
poor	1.0	0.0
average	21.0	28.0
good	67.0	61.0
very good	11.0	11.0
Maternal education level		
primary/lower secondary	1.0	2.0
basic vocational	8.0	4.0
secondary	42.0	57.0
higher	49.0	37.0
Paternal education level		
primary/lower secondary	3.0	5.1
basic vocational	20.2	16.2
secondary	43.4	54.5
higher	33.3	24.2
Parental nutrition knowledge		
insufficient	0.0	0.0
sufficient	5.0	8.0
good	69.0	77.0
very good	26.0	15.0
Child-pHDS category^2^		
low	48.0	45.0
moderate	39.0	37.0
high	13.0	18.0
Child-nHDS category^3^		
low	51.0	47.0
moderate	38.0	36.0
high	11.0	17.0
Child-UPFs category^4^		
low	58.0	45.0
moderate	27.0	37.0
high	15.0	18.0
Physical activity level compared to peers		
similar	84.0	75.0
lower	0.0	5.0
higher	16.0	20.0
BMI-for-age z-score^1,5^	*n* = 95 0.17 (1.58)	*n* = 99 -0.07 (1.52)
underweight	11.6	9.1
normal weight	78.9	80.8
overweight	7.4	9.1
obesity	2.1	1.0

^1^mean values and standard deviations (SD)

^2^Child-pHDS—Children’s pro-Healthy Diet Score (low: 0–2 points, moderate: 3–5 points, high: 6–7 points)

^3^Child-nHDS—Children’s non-Healthy Diet Score (low: 0–3 points, moderate: 4–7 points, high: 8–10 points)

^4^Child-UPFs—Children’s Ultra-Processed Food Score (low: 0–3 points, moderate: 4–7 points, high: 8–10 points)

^5^BMI-for-age z-scores—the age-specific Body Mass Index calculated using measured height and weight and assessed in categories according to the WHO standards [34]

### Reproducibility of food consumption frequency

The mean consumption frequency (times/day) of most food items assessed in both administrations of the 85-item Child-FFQ (test vs. retest) was not significantly different: 50/85 for children aged 13–36 months and 55/85 for children aged 37–72 months (Table 2).

**Table 2:** Mean values and standard deviations (SD) of the diet quality scores and food consumption frequency in test and retest of the 85-item Child-FFQ by children’s age groups

FFQ item no.	Diet quality scores/ Food items	Children aged 13–36 months (*n* = 100)			Children aged 37–72 months (*n* = 100)		
		Test	Retest	*p*	Test	Retest	*p*
		Mean (SD)			Mean (SD)		
	Diet quality scores (in points)						
	Child-pHDS ^1^	2.8 (2.0)		0.107	3.2 (2.1)	3.3 (1.9)	0.033
	Child-nHDS ^2^	3.4 (2.8)		0.001	4.3 (2.5)	4.6 (2.3)	0.001
	Child-UPFs ^3^	3.4 (2.9)		< 0.001	4.3 (2.6)	4.8 (2.6)	< 0.001
	Food consumption frequency (times/day)^4^						
1	White bread	0.80 (0.64)		0.152	1.12 (0.70)	1.10 (0.71)	0.636
2	Wholemeal bread	0.21 (0.35)		0.051	0.40 (0.52)	0.41 (0.50)	0.592
3	Rice wafers, crispbread	0.15 (0.23)		0.473	0.20 (0.29)	0.21 (0.30)	0.532
4	White rice	0.18 (0.25)		0.011	0.23 (0.30)	0.28 (0.37)	0.014
5	Brown rice	0.07 (0.23)		0.003	0.09 (0.20)	0.11 (0.21)	0.005
6	White pasta	0.25 (0.29)		0.030	0.32 (0.34)	0.34 (0.35)	0.159
7	Whole-grain pasta	0.09 (0.24)		0.021	0.14 (0.22)	0.17 (0.26)	0.021
8	Groats	0.18 (0.27)		0.189	0.21 (0.25)	0.24 (0.36)	0.414
9	Flavoured breakfast cereals	0.13 (0.23)		0.025	0.30 (0.28)	0.41 (0.52)	0.003
10	Corn flakes (unsweetened)	0.13 (0.20)		0.276	0.24 (0.26)	0.28 (0.31)	0.098
11	Natural cereals (e.g. oat flakes)	0.13 (0.21)		0.915	0.18 (0.24)	0.19 (0.25)	0.411
12	Cooked flour/potato-based dishes (e.g. dumplings)	0.24 (0.26)		0.081	0.26 (0.30)	0.32 (0.41)	0.064
13	Fried flour-based dishes (e.g. pancakes)	0.20 (0.22)		0.198	0.27 (0.29)	0.28 (0.30)	0.887
14	Fast food (excluding French fries)	0.03 (0.09)		0.086	0.07 (0.15)	0.08 (0.14)	0.102
15	Whole milk	0.16 (0.33)		0.212	0.37 (0.49)	0.40 (0.54)	0.180
16	Semi-skimmed milk	0.14 (0.34)		0.592	0.29 (0.38)	0.32 (0.50)	0.424
17	Skimmed milk	0.01 (0.06)		0.276	0.08 (0.27)	0.11 (0.36)	0.015
18	Flavoured milk beverages (e.g. cocoa)	0.03 (0.12)		0.066	0.10 (0.21)	0.15 (0.38)	0.013
19	Flavoured condensed milk	0.00 (0.01)		0.038	0.02 (0.10)	0.05 (0.18)	0.002
20	Goat milk	0.03 (0.20)		0.002	0.03 (0.15)	0.04 (0.23)	0.041
21	Natural yoghurt, kefir, buttermilk	0.20 (0.31)		0.254	0.21 (0.32)	0.26 (0.41)	0.027
22	Flavoured yoghurt, buttermilk	0.21 (0.25)		0.068	0.31 (0.30)	0.33 (0.38)	0.792
23	Cottage cheese and other unsweetened cheeses	0.19 (0.23)		0.189	0.23 (0.26)	0.24 (0.27)	0.389
24	Flavoured homogenised cheeses	0.24 (0.24)		0.043	0.32 (0.31)	0.32 (0.31)	0.725
25	Dairy desserts (e.g. pudding)	0.14 (0.20)		0.053	0.20 (0.23)	0.24 (0.31)	0.131
26	Ice-cream	0.04 (0.10)		0.324	0.14 (0.21)	0.17 (0.25)	0.035
27	Cheese	0.19 (0.26)		0.596	0.35 (0.33)	0.40 (0.42)	0.079
28	White meat	0.54 (0.29)		0.407	0.56 (0.34)	0.58 (0.41)	0.617
29	Red meat	0.19 (0.28)		0.017	0.26 (0.30)	0.28 (0.36)	0.273
30	High-quality cold cuts	0.50 (0.47)		0.021	0.63 (0.46)	0.69 (0.59)	0.212
31	Sausages, pates	0.31 (0.35)		0.693	0.46 (0.45)	0.47 (0.50)	0.930
32	Fresh/frozen fish	0.14 (0.27)		0.322	0.15 (0.31)	0.20 (0.42)	0.095
33	Smoked fish	0.01 (0.03)		0.002	0.07 (0.23)	0.09 (0.30)	0.014
34	Canned fish	0.02 (0.08)		0.015	0.06 (0.24)	0.08 (0.26)	0.007
35	Seafood	0.01 (0.02)		0.010	0.03 (0.13)	0.04 (0.12)	0.046
36	Eggs	0.38 (0.30)		0.652	0.42 (0.37)	0.46 (0.46)	0.407
37	Vegetables (fresh/frozen)	0.69 (0.60)		0.362	0.80 (0.66)	0.79 (0.67)	0.597
38	Vegetables (e.g. boiled, including soups)	0.53 (0.38)		0.895	0.49 (0.36)	0.55 (0.51)	0.075
39	Vegetable pastes	0.08 (0.18)		0.034	0.12 (0.21)	0.16 (0.25)	0.014
40	Pickled vegetables	0.13 (0.28)		0.129	0.18 (0.29)	0.24 (0.40)	0.001
41	Pulses and pulse-based dishes	0.09 (0.19)		0.022	0.14 (0.27)	0.17 (0.31)	0.029
42	Potatoes (excluding fries)	0.46 (0.33)		0.689	0.56 (0.32)	0.56 (0.41)	0.985
43	French fries	0.08 (0.24)		0.021	0.17 (0.28)	0.19 (0.30)	0.084
44	Ketchup	0.07 (0.16)		0.011	0.30 (0.42)	0.31 (0.43)	0.397
45	Fruit (fresh/frozen)	0.79 (0.62)		0.206	0.79 (0.69)	0.77 (0.70)	0.490
46	Fruit mousse	0.40 (0.47)		0.590	0.29 (0.34)	0.33 (0.42)	0.097
47	Canned fruit	0.04 (0.12)		0.003	0.11 (0.27)	0.11 (0.20)	0.347
48	Dried fruit	0.05 (0.14)		0.005	0.12 (0.28)	0.12 (0.27)	0.144
49	Nuts, seeds	0.05 (0.13)		0.016	0.14 (0.29)	0.16 (0.37)	0.694
50	Peanut butter (unsweetened)	0.03 (0.10)		0.269	0.12 (0.30)	0.14 (0.31)	0.027
51	Butter	0.93 (0.71)		0.098	1.17 (0.68)	1.15 (0.68)	0.530
52	Cream	0.23 (0.32)		0.004	0.31 (0.33)	0.36 (0.50)	0.452
53	Margarines, butter and margarine mixes	0.13 (0.39)		0.506	0.17 (0.43)	0.20 (0.49)	0.053
54	Olive oil	0.15 (0.24)		0.453	0.23 (0.35)	0.29 (0.47)	0.005
55	Vegetable oils	0.19 (0.25)		0.275	0.28 (0.36)	0.31 (0.40)	0.076
56	Mayonnaise	0.03 (0.12)		0.036	0.11 (0.31)	0.13 (0.36)	0.176
57	Lard	0.02 (0.10)		0.006	0.05 (0.23)	0.07 (0.24)	0.001
58	Herbal seasoning	0.49 (0.47)		0.015	0.59 (0.48)	0.61 (0.53)	0.718
59	Salty condiments with vegetables (e.g. bouillon cubes)	0.17 (0.33)		0.061	0.27 (0.43)	0.32 (0.48)	0.005
60	Instant soups and sauces	0.09 (0.27)		0.097	0.17 (0.36)	0.19 (0.36)	0.130
61	Sugar	0.32 (0.50)		0.088	0.59 (0.55)	0.65 (0.63)	0.115
62	Honey	0.21 (0.27)		0.018	0.35 (0.43)	0.39 (0.49)	0.031
63	Fruit syrups	0.10 (0.27)		0.210	0.18 (0.31)	0.19 (0.32)	0.328
64	Jam, marmalade	0.12 (0.18)		0.784	0.24 (0.26)	0.26 (0.32)	0.403
65	Chocolate/chocolate-nut spreads	0.05 (0.12)		0.041	0.14 (0.21)	0.14 (0.21)	0.422
66	Chocolate, chocolate bars	0.09 (0.17)		0.091	0.17 (0.21)	0.21 (0.26)	0.049
67	Candies	0.11 (0.25)		0.357	0.22 (0.29)	0.23 (0.37)	0.554
68	Sponge cookies, biscuits	0.33 (0.34)		0.008	0.29 (0.26)	0.33 (0.36)	0.169
69	Other cookies	0.12 (0.18)		0.262	0.18 (0.21)	0.21 (0.26)	0.084
70	Cakes and pastries	0.11 (0.16)		0.037	0.24 (0.24)	0.30 (0.36)	0.054
71	Corn crisps	0.35 (0.42)		0.098	0.30 (0.34)	0.31 (0.36)	0.440
72	Salty snacks	0.07 (0.15)		0.010	0.18 (0.22)	0.25 (0.40)	0.015
73	Mineral/spring water	1.33 (0.83)		0.041	1.34 (0.80)	1.38 (0.78)	0.181
74	Flavoured water (commercial)	0.10 (0.28)		0.062	0.20 (0.47)	0.19 (0.43)	0.553
75	Sweetened tea	0.34 (0.56)		0.609	0.49 (0.60)	0.51 (0.62)	0.466
76	Unsweetened tea	0.27 (0.52)		0.022	0.25 (0.48)	0.27 (0.47)	0.529
77	Fruit compotes (sweetened)	0.14 (0.24)		0.231	0.28 (0.43)	0.29 (0.43)	0.918
78	Homemade/freshly squeezed fruit juices	0.13 (0.28)		0.051	0.15 (0.24)	0.22 (0.41)	0.003
79	Fruit juices (commercial)	0.25 (0.38)		0.667	0.43 (0.46)	0.40 (0.42)	0.273
80	Homemade/freshly squeezed vegetable and vegetable-fruit juices	0.08 (0.16)		0.035	0.08 (0.19)	0.11 (0.28)	0.020
81	Vegetable and vegetable-fruit juices (commercial)	0.09 (0.17)		0.959	0.14 (0.24)	0.16 (0.25)	0.041
82	Plant-based beverages	0.02 (0.08)		0.002	0.04 (0.14)	0.05 (0.15)	0.001
83	Smoothies/cocktails (e.g. vegetable-fruit, milk-fruit)	0.03 (0.09)		0.003	0.06 (0.15)	0.09 (0.24)	0.020
84	Sugar-sweetened soft drinks	0.04 (0.15)		0.506	0.12 (0.29)	0.16 (0.33)	0.025
85	Diet soft drinks	0.03 (0.14)		0.011	0.06 (0.19)	0.07 (0.20)	0.021

*SD S*tandard deviation, *p S*ignificance level of Wilcoxon signed-rank test

^1^Child-pHDS—Children’s pro-Healthy Diet Score (range: 0–7 points)

^2^Child-nHDS—Children’s non-Healthy Diet Score (range: 0–10 points)

^3^Child-UPFs—Children’s Ultra-Processed Food Score (range: 0–10 points); NA—not applied

^4^daily consumption frequency (range: 0–2 times/day)

The total classification agreement for food consumption frequency was above 50% for all food items in the three analysed age groups of children (Table 3). Among children aged 13–36 months, the total average classification agreement for food consumption frequency was 87.6%, ranging from 74.0% (herbal seasoning) to 97.0% (skimmed milk) (Table 3). Misclassification by one frequency category was on average 8.1% and ranged from 1.0% (skimmed milk) to 17.0% (herbal seasoning; sponge cookies/biscuits), while misclassification by at least two frequency categories was on average 4.4% and ranged from 0% (sausages/pates) to 9.0% (wholemeal bread; natural yogurt/kefir/buttermilk; herbal seasoning). The kappa statistic was on average 0.78, ranging from 0.46 (diet soft drinks) to 0.92 (vegetable pastes) (Table 3).

**Table 3:** Classification agreement (%) and kappa statistics for the diet quality scores and food consumption frequency in test and retest of the 85-item Child-FFQ by children’s age groups

FFQ item no.	Diet quality scores/ Food items	Children aged 13–36 months (*n* = 100)				Children aged 37–72 months (*n* = 100)			
		Total Agreement	Misclassification		Kappa	Total Agreement	Misclassification		Kappa
			± 1	± 2 or more categories			± 1	± 2 or more categories	
	Diet quality scores								
	Child-pHDS ^1^	77.0	23.0	0.0	0.62	84.0	16.0	0.0	0.75
	Child-nHDS ^2^	92.0	8.0	0.0	0.86	79.0	21.0	0.0	0.67
	Child-UPFs ^3^	86.0	14.0	0.0	0.77	81.0	19.0	0.0	0.70
	Food consumption frequency^4^								
	Mean value of all items	87.6	8.1	4.4	0.78	86.1	9.2	4.6	0.79
1	White bread	83.0	10.0	7.0	0.78	84.0	13.0	3.0	0.78
2	Wholemeal bread	82.0	9.0	9.0	0.75	89.0	8.0	3.0	0.86
3	Rice wafers, crispbread	88.0	10.0	2.0	0.83	89.0	7.0	4.0	0.86
4	White rice	88.0	8.0	4.0	0.83	89.0	6.0	5.0	0.84
5	Brown rice	89.0	8.0	3.0	0.80	90.0	5.0	5.0	0.85
6	White pasta	88.0	7.0	5.0	0.84	91.0	8.0	1.0	0.87
7	Whole-grain pasta	90.0	6.0	4.0	0.85	89.0	9.0	2.0	0.85
8	Groats	88.0	10.0	2.0	0.84	81.0	17.0	2.0	0.75
9	Flavoured breakfast cereals	89.0	4.0	7.0	0.83	79.0	13.0	8.0	0.73
10	Corn flakes (unsweetened)	87.0	10.0	3.0	0.82	86.0	9.0	5.0	0.82
11	Natural cereals (e.g. oat flakes)	94.0	4.0	2.0	0.91	90.0	8.0	2.0	0.87
12	Cooked flour/potato-based dishes (e.g. dumplings)	85.0	11.0	4.0	0.80	84.0	10.0	6.0	0.78
13	Fried flour-based dishes (e.g. pancakes)	85.0	9.0	6.0	0.80	92.0	7.0	1.0	0.89
14	Fast food (excluding French fries)	93.0	3.0	4.0	0.82	85.0	12.0	3.0	0.76
15	Whole milk	91.0	5.0	4.0	0.84	89.0	9.0	2.0	0.86
16	Semi-skimmed milk	89.0	7.0	4.0	0.80	85.0	11.0	4.0	0.80
17	Skimmed milk	97.0	1.0	2.0	0.74	87.0	8.0	5.0	0.70
18	Flavoured milk beverages (e.g. cocoa)	96.0	2.0	2.0	0.87	86.0	9.0	5.0	0.78
19	Flavoured condensed milk	95.0	2.0	3.0	0.60	88.0	5.0	7.0	0.66
20	Goat milk	89.0	7.0	4.0	0.56	95.0	3.0	2.0	0.71
21	Natural yoghurt, kefir, buttermilk	83.0	8.0	9.0	0.77	84.0	10.0	6.0	0.79
22	Flavoured yoghurt, buttermilk	85.0	9.0	6.0	0.80	82.0	15.0	3.0	0.76
23	Cottage cheese and other unsweetened cheeses	83.0	12.0	5.0	0.78	93.0	6.0	1.0	0.91
24	Flavoured homogenised cheeses	86.0	8.0	6.0	0.81	82.0	11.0	7.0	0.74
25	Dairy desserts (e.g. pudding)	93.0	5.0	2.0	0.90	84.0	11.0	5.0	0.79
26	Ice-cream	92.0	5.0	3.0	0.86	85.0	9.0	6.0	0.77
27	Cheese	87.0	8.0	5.0	0.82	83.0	13.0	4.0	0.77
28	White meat	78.0	16.0	6.0	0.60	85.0	12.0	3.0	0.71
29	Red meat	86.0	10.0	4.0	0.82	87.0	9.0	4.0	0.83
30	High-quality cold cuts	90.0	6.0	4.0	0.87	79.0	13.0	8.0	0.72
31	Sausages, pates	87.0	13.0	0.0	0.83	85.0	12.0	3.0	0.79
32	Fresh/frozen fish	87.0	12.0	1.0	0.82	88.0	9.0	3.0	0.83
33	Smoked fish	85.0	7.0	8.0	0.58	90.0	6.0	4.0	0.82
34	Canned fish	89.0	6.0	5.0	0.60	91.0	5.0	4.0	0.76
35	Seafood	92.0	5.0	3.0	0.62	89.0	5.0	6.0	0.63
36	Eggs	81.0	13.0	6.0	0.71	77.0	15.0	8.0	0.67
37	Vegetables (fresh/frozen)	83.0	12.0	5.0	0.78	82.0	14.0	4.0	0.77
38	Vegetables (e.g. boiled, including soups)	84.0	11.0	5.0	0.77	78.0	18.0	4.0	0.70
39	Vegetable pastes	95.0	4.0	1.0	0.92	82.0	11.0	7.0	0.74
40	Pickled vegetables	89.0	6.0	5.0	0.84	85.0	7.0	8.0	0.80
41	Pulses and pulse-based dishes	88.0	8.0	4.0	0.81	84.0	11.0	5.0	0.78
42	Potatoes (excluding fries)	82.0	15.0	3.0	0.71	79.0	18.0	3.0	0.64
43	French fries	88.0	5.0	7.0	0.81	91.0	7.0	2.0	0.88
44	Ketchup	90.0	3.0	7.0	0.82	89.0	6.0	5.0	0.86
45	Fruit (fresh/frozen)	86.0	13.0	1.0	0.82	85.0	11.0	4.0	0.81
46	Fruit mousse	91.0	5.0	4.0	0.89	89.0	7.0	4.0	0.86
47	Canned fruit	89.0	7.0	4.0	0.78	85.0	10.0	5.0	0.78
48	Dried fruit	86.0	8.0	6.0	0.71	88.0	6.0	6.0	0.82
49	Nuts, seeds	87.0	7.0	6.0	0.73	88.0	8.0	4.0	0.83
50	Peanut butter (unsweetened)	96.0	2.0	2.0	0.86	90.0	7.0	3.0	0.83
51	Butter	87.0	10.0	3.0	0.83	85.0	8.0	7.0	0.79
52	Cream	88.0	5.0	7.0	0.84	81.0	16.0	3.0	0.75
53	Margarines, butter and margarine mixes	90.0	6.0	4.0	0.77	86.0	7.0	7.0	0.79
54	Olive oil	88.0	6.0	6.0	0.84	90.0	6.0	4.0	0.87
55	Vegetable oils	83.0	14.0	3.0	0.77	85.0	8.0	7.0	0.80
56	Mayonnaise	91.0	5.0	4.0	0.75	93.0	2.0	5.0	0.90
57	Lard	91.0	6.0	3.0	0.56	87.0	10.0	3.0	0.71
58	Herbal seasoning	74.0	17.0	9.0	0.67	83.0	13.0	4.0	0.77
59	Salty condiments with vegetables (e.g. bouillon cubes)	86.0	8.0	6.0	0.78	89.0	5.0	6.0	0.86
60	Instant soups and sauces	89.0	7.0	4.0	0.79	87.0	6.0	7.0	0.82
61	Sugar	80.0	13.0	7.0	0.74	85.0	9.0	6.0	0.81
62	Honey	88.0	6.0	6.0	0.84	82.0	14.0	4.0	0.77
63	Fruit syrups	91.0	7.0	2.0	0.84	90.0	8.0	2.0	0.86
64	Jam, marmalade	89.0	9.0	2.0	0.85	82.0	13.0	5.0	0.75
65	Chocolate/chocolate-nut spreads	90.0	6.0	4.0	0.81	89.0	6.0	5.0	0.85
66	Chocolate, chocolate bars	90.0	7.0	3.0	0.85	87.0	8.0	5.0	0.82
67	Candies	90.0	6.0	4.0	0.85	81.0	15.0	4.0	0.74
68	Sponge cookies, biscuits	81.0	17.0	2.0	0.75	87.0	8.0	5.0	0.82
69	Other cookies	85.0	12.0	3.0	0.79	83.0	13.0	4.0	0.77
70	Cakes and pastries	85.0	7.0	8.0	0.79	79.0	14.0	7.0	0.70
71	Corn crisps	80.0	13.0	7.0	0.75	85.0	10.0	5.0	0.80
72	Salty snacks	87.0	6.0	7.0	0.79	87.0	7.0	6.0	0.82
73	Mineral/spring water	85.0	11.0	4.0	0.77	89.0	6.0	5.0	0.82
74	Flavoured water (commercial)	83.0	12.0	5.0	0.66	93.0	4.0	3.0	0.90
75	Sweetened tea	93.0	5.0	2.0	0.90	86.0	11.0	3.0	0.83
76	Unsweetened tea	88.0	10.0	2.0	0.81	85.0	10.0	5.0	0.80
77	Fruit compotes (sweetened)	81.0	14.0	5.0	0.72	90.0	7.0	3.0	0.87
78	Homemade/freshly squeezed fruit juices	93.0	3.0	4.0	0.89	83.0	9.0	8.0	0.77
79	Fruit juices (commercial)	85.0	14.0	1.0	0.80	81.0	12.0	7.0	0.76
80	Homemade/freshly squeezed vegetable and vegetable-fruit juices	87.0	8.0	5.0	0.79	89.0	6.0	5.0	0.82
81	Vegetable and vegetable-fruit juices (commercial)	90.0	9.0	1.0	0.84	83.0	9.0	8.0	0.75
82	Plant-based beverages	88.0	4.0	8.0	0.60	87.0	10.0	3.0	0.72
83	Smoothies/cocktails (e.g. vegetable-fruit, milk-fruit)	86.0	6.0	8.0	0.64	91.0	3.0	6.0	0.84
84	Sugar-sweetened soft drinks	91.0	4.0	5.0	0.72	88.0	4.0	8.0	0.81
85	Diet soft drinks	85.0	10.0	5.0	0.46	86.0	9.0	5.0	0.70

^1^Child-pHDS—Children’s pro-Healthy Diet Score (in 3 categories: low 0–2 points, moderate 3–5 points, high 6–7 points)

^2^Child-nHDS—Children’s non-Healthy Diet Score (in 3 categories: low 0–3 points, moderate 4–7 points, high 8–10 points)

^3^Child-UPFs—Children’s Ultra-Processed Food Score (in 3 categories: low 0–3 points, moderate 4–7 points, high 8–10 points)

^4^food consumption frequency (in 6 categories)

Among children aged 37–72 months, the total classification agreement for food consumption frequency was on average 86.1%, ranging from 77.0% (eggs) to 95.0% (goat milk) (Table 3). Misclassification by one frequency category was on average 9.2% and ranged from 2.0% (mayonnaise) to 18.0% (boiled vegetables; potatoes), while misclassification by at least two frequency categories was on average 4.6% and ranged from 1.0% (white pasta; fried flour-based dishes; cottage cheese and other unsweetened cheeses) to 8.0% (7 food items). The kappa statistic was on average 0.79, ranging from 0.63 (seafood) to 0.91 (cottage cheese and other unsweetened cheeses) (Table 3).

### Reproducibility of diet quality scores

The mean values of the child-pHDS assessed based on the dietary data from test and retest of the 85-item Child-FFQ were not significantly different in children aged 13–36 months (Table 2). The total classification agreement was above 50% for both diet quality scores in the two children’s age groups for which they were developed (Table 3). The total classification agreement was 77.0% for child-pHDS, 92.0% for child-nHDS and 86.0% for child-UPFs in children aged 13–36 months, while 84.0%, 79.0% and 81.0% in children aged 37–72 months respectively. In general, misclassification by one category ranged from 8.0% to 23.0% of children, while misclassification by two categories was not found in any child from both age groups.

The kappa statistic was above 0.40 for both diet quality scores in the two analyzed children’s age groups (Table 3). The kappa statistic was 0.62 for child-pHDS, 0.86 for child-nHDS and 0.77 for child-UPFs in children aged 13–36 months, while 0.75, 0.67 and 0.70 in children aged 37–72 months, respectively.

The Spearman’s correlation coefficient for the diet quality scores assessed based on the dietary data from test and retest of the 85-item Child-FFQ ranged from 0.889 (child-pHDS in children aged 13–36 months) to 0.947 (child-nHDS in children aged 13–36 months), and the ICC ranged from 0.886 to 0.950, respectively (Table 4).

**Table 4:** Spearman’s correlation coefficient and intraclass correlation coefficient for the diet quality scores in test and retest of the 85-item Child-FFQ by children’s age groups

Diet quality scores	Children aged 13–36 months (*n* = 100)					Children aged 37–72 months (*n* = 100)				
	*r*	*p*	ICC	95% CI	*p*	*r*	*p*	ICC	95% CI	*p*
Child-pHDS ^1^	0.889	< 0.001	0.886	0.836–0.922	< 0.001	0.906	< 0.001	0.912	0.871–0.940	< 0.001
Child-nHDS ^2^	0.947	< 0.001	0.950	0.917–0.968	< 0.001	0.914	< 0.001	0.913	0.860–0.944	< 0.001
Child-UPFs ^3^	0.924	< 0.001	0.914	0.744–0.960	< 0.001	0.929	< 0.001	0.918	0.837–0.954	< 0.001

*r* Spearman’s correlation coefficient, *ICC* Intraclass correlation coefficient, *95% CI* confidence interval for ICC; p – significance level

^1^Child-pHDS—Children’s pro-Healthy Diet Score (range: 0–7 points)

^2^Child-nHDS—Children’s non-Healthy Diet Score (range: 0–10 points)

^3^Child-UPFs—Children’s Ultra-Processed Food Score (range: 0–10 points)

**Fig. 1:**
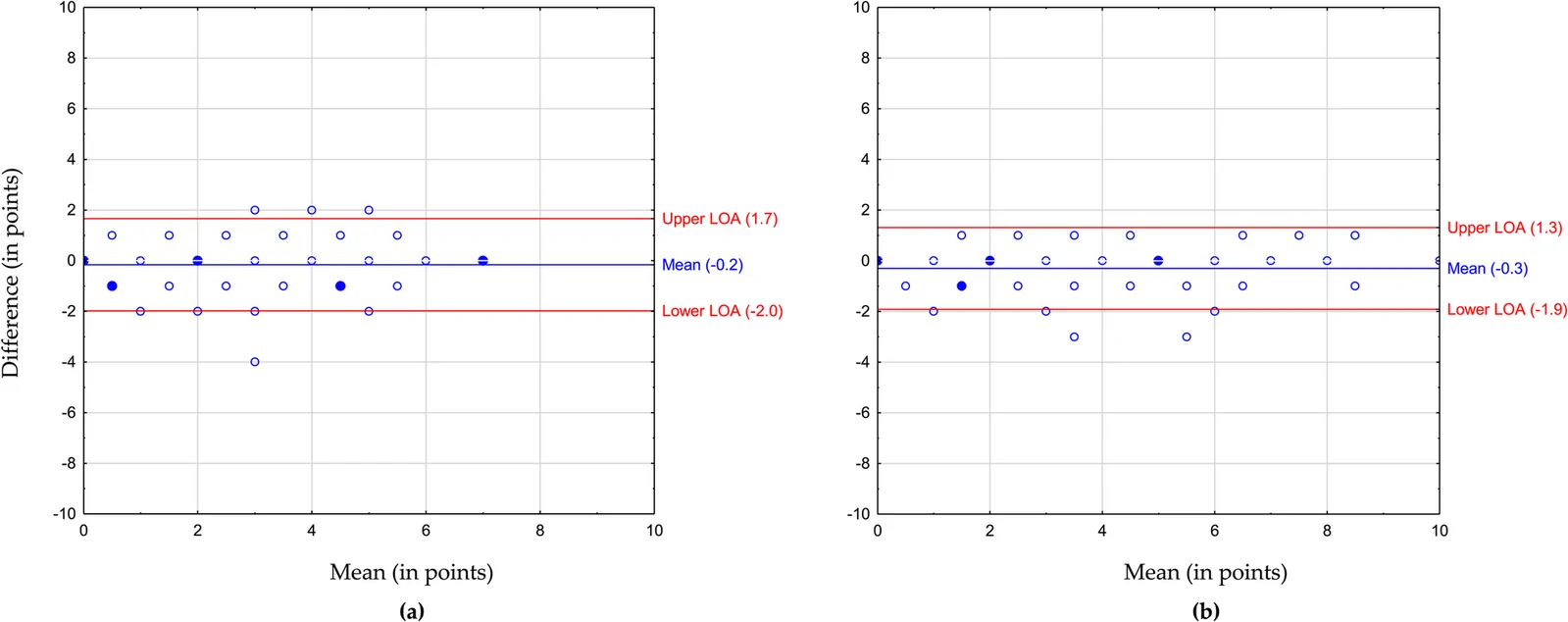
The child-pHDS: Bland–Altman plots for test and retest in children aged 13–36 months **a** and children aged 37–72 months **b**. Mean—mean difference between the test and retest of the questionnaire (blue solid line). LOA—95% limits of agreement between the test and retest of the questionnaire (red solid lines)

**Fig. 2:**
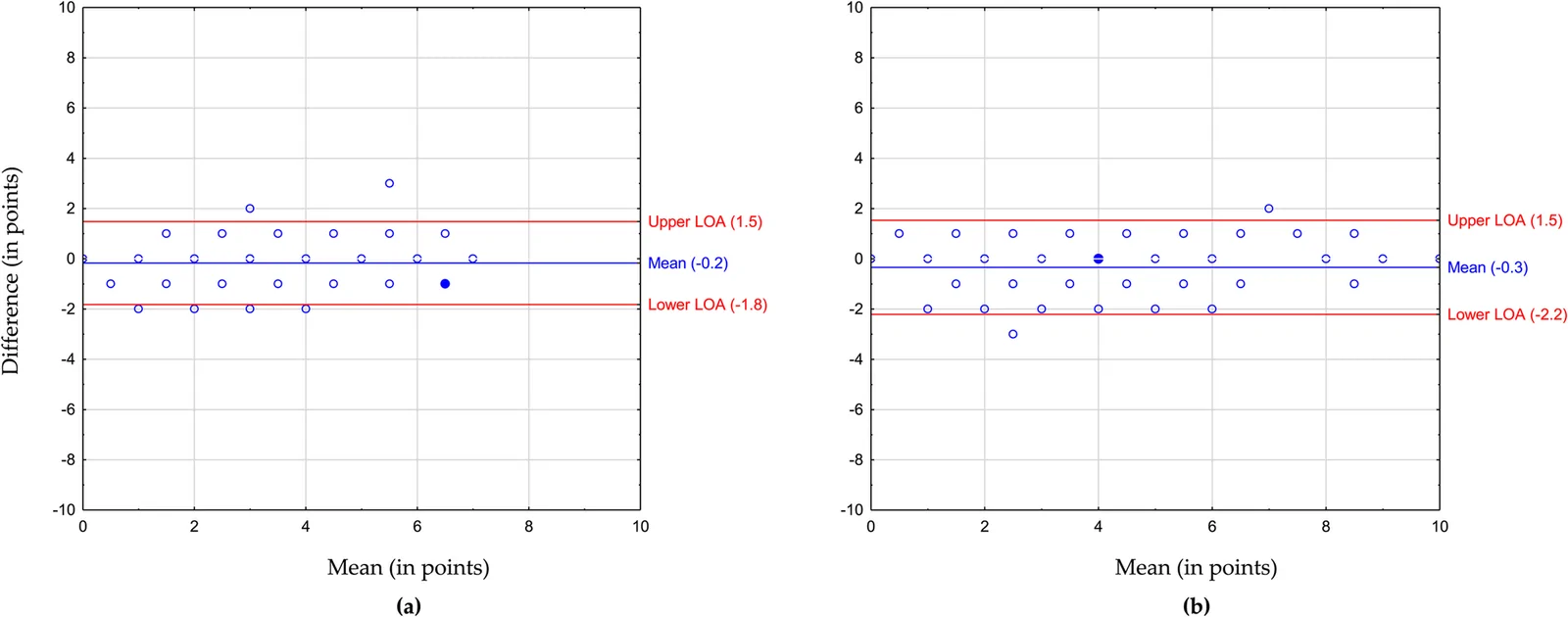
The child-nHDS: Bland–Altman plots for test and retest in children aged 13–36 months **a** and children aged 37–72 months **b**. Mean—mean difference between the test and retest of the questionnaire (blue solid line). LOA—95% limits of agreement between the test and retest of the questionnaire (red solid lines)

**Fig. 3:**
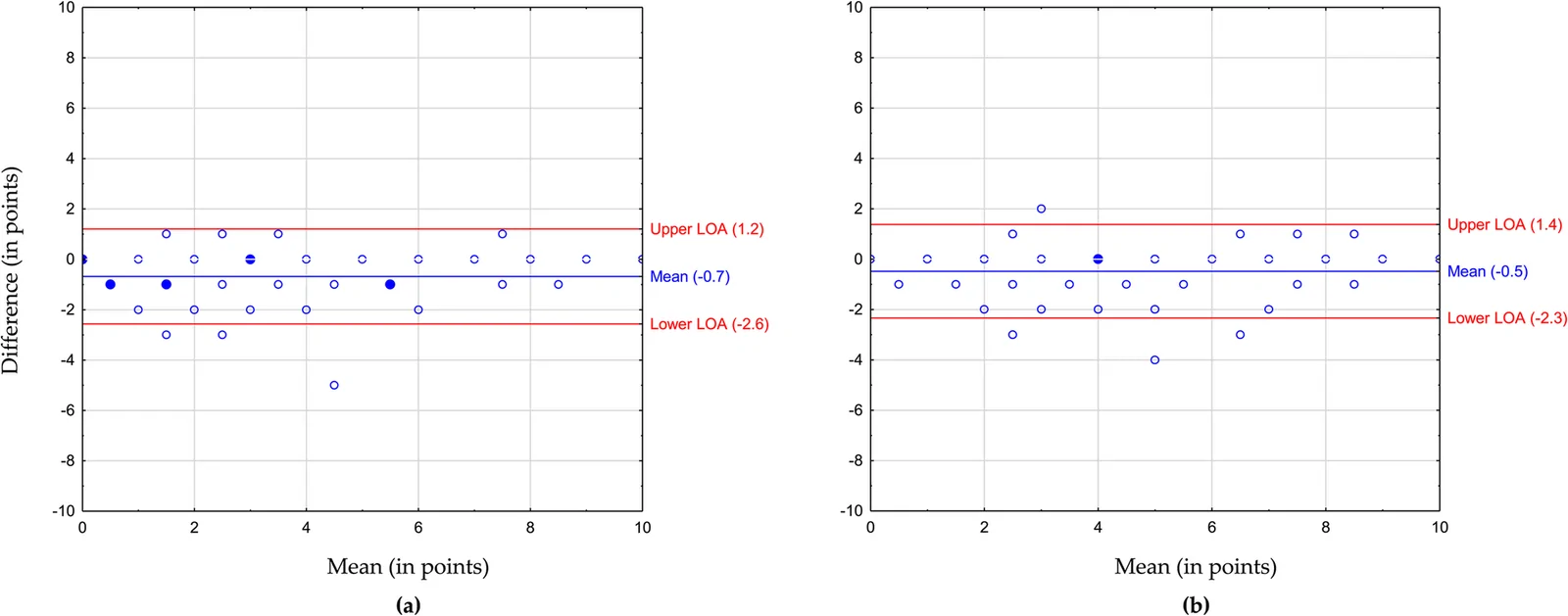
The child-UPFs: Bland–Altman plots for test and retest in children aged 13–36 months **a** and children aged 37–72 months **b**. Mean—mean difference between the test and retest of the questionnaire (blue solid line). LOA—95% limits of agreement between the test and retest of the questionnaire (red solid lines)

The Bland–Altman plots showed mean difference ranged from − 0.2 points (both child-pHDS and child-nHDS in children aged 13–36 months) to -0.7 points (child-UPFs in children aged 13–36 months) (Figs. 1, 2 and 3).

## Discussion

The current study showed generally good test–retest reproducibility for the 85-item Child-FFQ, and good to excellent for the newly developed diet quality scores among children aged 13–72 months. Regarding food consumption frequency, the classification agreement was high across both age groups, and the kappa statistic indicating moderate to excellent agreement. Considering the diet quality scores indices, the reproducibility was characterized by high classification agreement, good to excellent kappa statistics as well as the reliability assessed with the intraclass correlation coefficient (ICC). In the Bland-Altman analysis, a small negative shift and moderately wide limits of agreement were observed for the analysed diet quality scores.

Overall, the 85-item Child-FFQ demonstrated good test–retest reproducibility in Polish toddlers and preschool-age children. The vast majority of food items showed no statistically significant difference in reported consumption frequency between the first and second administration of the FFQ. Considering the group level, the mean consumption frequency for almost two-thirds of the food items (50–55 of 85 items) was not statistically different between the test and retest of the FFQ. The food items that showed differences in the mean consumption frequency tended to be those eaten irregularly or in highly variable amounts, i.e., foods/ dishes which could reflect either random variation or inaccuracies in recall. Importantly, the magnitude of these differences was generally minor, and there was no systematic bias toward higher or lower consumption frequency in retest. A possible factor that may affect the observed findings is the administration of the 85-item Child-FFQ by an interviewer, which might improve correlation compared with self-administration by parents/proxies [4, 21]. The overall reproducibility of the 85-item Child-FFQ was good, consistent with findings from similar studies worldwide and, in some cases, better [7, 11, 14–16, 35]. Differences in specific metrics across studies must also be interpreted in consideration of the test-retest interval, administration method, sample characteristics, and statistical measures used, e.g., most studies reported Pearson/Spearman correlations and ICC.

Observed consistency in mean food-consumption frequency was supported by cross-classification analysis. Across age groups, the total classification agreement for food consumption frequency, i.e., reporting the same frequency category in both the test and retest, ranged from 74% to 97%. Cases of significant misclassification were rare. Infrequent misclassification by at least two frequency categories concerned ≤ 9% respondents. The kappa statistic indicated moderate to excellent agreement in reported categories of food consumption frequency between the two administrations of the FFQ. Kappa statistic was acceptable, i.e., above 0.40, for all food items across all age groups. In other studies, lower FFQ reproducibility was typically found for snacks and beverages, particularly soft drinks [36–38]. Rarely consumed foods typically yield lower kappa values despite minimal absolute differences, due to kappa’s statistical sensitivity to distribution [39]. Overall, our results indicate that parents provided internally consistent responses on the food consumption frequency of their children, and any random errors in recall were negligible.

The reproducibility of the diet quality scores based on the FFQ data was also good, especially on the individual level. On a group level, we observed no significant difference in mean score values between test and retest for the child-pHDS in children aged 13–36 months. The total classification agreement for each diet quality score, analyzed as a variable with three categories, ranged from 77% to 92%. No child was misclassified by more than one category on retest for any of the diet quality scores, which implies that, for example, a child categorized as having a low child-pHDS at the test was never classified as having a high child-pHDS at the retest, or vice versa. The kappa statistic for the diet quality scores corresponded from good to excellent agreement between both administrations of the FFQ. Very high test–retest Spearman correlations and ICCs confirmed the reproducibility of the diet quality scores. This indicates that the 85-item Child-FFQ can reproducibly capture habitual consumption frequencies of foods among toddlers and preschool-age children. Furthermore, the diet quality scores developed to measure opposing dietary patterns among children aged 13–72 months are likewise reliable. Several other studies conducted among toddlers and preschool-age children have validated FFQ-derived diet quality scores, often by comparing the FFQ-based score to food records or repeated 24-hour dietary recalls [40, 41], by testing construct validity against nutrient profiles [42–44] or health/behavior indicators [45, 46]. Regarding the reproducibility of an FFQ-based diet quality score, Huybrechts et al. [40] reported a weighted kappa of 0.61 and a correlation coefficient of 0.88 for the total diet quality index in preschool children, which is comparable to our results. Watson et al. [14] reported a mean intra-class correlation coefficient of 0.71 (range, 0.65–0.75) for the FFQ designed to assess nutrient intake and dietary patterns among toddlers aged 12–24 months.

The Bland–Altman analysis was used to assess the agreement between two measurements of dietary quality scores derived from the FFQ (test and retest). Negative mean bias values (ranging from − 0.2 to -0.7) indicate a small, systematic shift toward higher values in the retest. The largest bias was observed for child‑UPFs. Consequently, the results support using the tool for group-level comparisons and rankings. A moderately broad 95% LOA indicates that scores should be interpreted with caution in individual-level assessments. For both dietary quality scores and FFQs, small to moderate bias is frequently observed, but the LOAs tend to be wide, indicating that these tools are best suited for ranking and group-level comparisons, but less reliable for individual-level assessment [40, 47, 48].

In recent years, there has been growing interest in developing and validating practical indices of UPF intake in young children’s diets. The approaches included evaluating the proportion of dietary energy from UPFs [19, 49] and developing scoring systems of UPF consumption frequency [48, 50]. Studies have shown that a healthy feeding pattern is associated with lower UPF consumption in children. Tracking of UPF intake can strengthen dietary assessments in this age group. Incorporating a UPF-focused diet quality score, like child-UPFs, allows researchers and practitioners to better identify young children with suboptimal diets and to compare results across studies and populations in this emerging field of nutritional epidemiology.

The 85-item Child-FFQ was developed based on the questionnaire previously used in the PITNUTS 2016 study, which included children aged 5–36 months [51], and further modified and refined by nutrition experts with experience in developing and validating FFQs among different population groups in Poland to ensure suitability for a wider age range (5–72 months) and contemporary dietary patterns. The strengths of the present study include its conduction under standardized administration conditions and random selection of 200 children for this FFQ reproducibility study from the nationally representative sample of 1,000 Polish children recruited to the PITNUTS 2024 study. The questionnaire was interviewer-administered, although it is more resource-intensive than using self-administered versions. These methodological conditions contributed to obtaining more reliable data. Next, several different statistical methods were used to analyze the data, which strengthened the conclusions.

However, during the interviewer-administered FFQ, parents acting as proxy reporters may have systematically under-reported certain “unhealthy” foods or over-reported “healthy” ones due to the potential social desirability bias, which is typical for self-report methods. Furthermore, the small negative bias observed particularly for the child-UPFs score aligns with the established literature showing that the first administration of a dietary questionnaire usually tends to yield higher results than subsequent ones [52]. For future longitudinal applications of the 85-item Child-FFQ, this systematic shift underscores that while the tool remains robust for ranking children’s diet quality, absolute frequencies of UPF consumption may be slightly underestimated over time due to a respondent learning effect or social desirability bias among parents acting as proxy reporters.

The present study assessed the test–retest reproducibility of the 85-item Child-FFQ but did not validate the questionnaire against biomarkers. The two-week interval was chosen carefully based on a review of previous studies on the reproducibility of the FFQs targeted to small children [22, 53–55]. This timeframe is long enough to prevent parents from simply recalling their previous answers, yet short enough to ensure that the children’s actual dietary behaviours did not undergo genuine developmental or seasonal changes. These changes could lower reliability scores, thereby underestimating the performance of the tool [4, 54]. Furthermore, the 85-item Child-FFQ is a qualitative food frequency questionnaire and does not collect portion size data; thus, it cannot be used to estimate absolute energy or nutrient intake. This FFQ was used in the PITNUTS 2024 study in parallel with a 3-day food record to ensure qualitative and quantitative evaluation of the diet and its quality [18]. The relationship between developed diet quality scores and the risk of inadequate nutrient intake estimated based on 3-day food records in the representative sample of Polish children have been published previously [56].

Regardless of the above issues, the 85-item Child-FFQ, developed for children aged 13–72 months, fills a gap in the library of Polish tools used to evaluate children’s habitual diet. Previously, only a few research tools intended for children and validated at least in terms of assessing test-retest reproducibility were available in Poland. For example, the Short-Form, Multicomponent Dietary Questionnaire to Assess Food Frequency Consumption, Nutrition Knowledge, and Lifestyle (SF-FFQ4PolishChildren^®^) was developed and tested for test-retest reproducibility in Polish children aged 6–10 years old and adolescents aged 11–15 years [22]. Other, a semi-quantitative FFQ was validated against 24-h dietary recalls in Polish children aged 3 years, showing overestimation of energy and nutrient intake by the FFQ [57].

The diet quality scores (child-pHDS, child-nHDS, child-UPFs), developed for children aged 13–72 months and based on dietary data collected using this FFQ, are useful for complex assessment of various dietary characteristics in early childhood. The cut-off points for each food group in the diet quality scores were based on median values of consumption frequency estimated separately for each age cohort from a representative, nationwide sample (PITNUTS 2024 study). This data-driven approach is well-established in literature concerning the development of diet quality scores and their utilization in nutritional epidemiology [58–60]. This methodology effectively differentiates lower and higher consumers relative to their national peers, providing a reliable solution especially when official pediatric recommendations regarding precise food group consumption frequencies for all analyzed food groups have not been established. Our results indicate that these diet quality scores are reproducible and can effectively identify subgroups of children with high adherence to these dietary patterns of concern, as well as detect differences in diet quality between groups of children. Despite the Cronbach’s alpha for the child-pHDS among younger children was marginally the recommended range (0.70–0.90), this version proved optimal among all tested variations, and its overall reliability was confirmed by all other metrics. Furthermore, utilizing these indices allows researchers to restrict the 85-item Child-FFQ exclusively to the components included in the diet quality scores, which has practical importance in study settings that require a simple, cost-effective, and low-burden method for dietary assessment.

While the 85-item Child-FFQ demonstrated good short-term stability, further longitudinal research is required to evaluate its responsiveness to long-term dietary shifts. Beyond the local context, the reproducibility findings of this newly developed FFQ can be highly valuable to other culturally similar countries, providing valuable methodological information on dietary assessment in early childhood.

## Conclusions

The newly developed 85-item Child-FFQ showed a good to excellent test-retest reproducibility. Therefore, it can be recommended as a reliable tool for assessing habitual food consumption frequency and evaluating diet quality, using indices derived from this FFQ (child-pHDS, child-nHDS, and child-UPFs), among Polish children aged 13–72 months. This tool has the potential to help monitor food consumption and diet quality variations over time, though this capacity needs to be validated through future longitudinal studies.

## Supplementary Information

Supplementary Material 1.

## Data Availability

The dataset analysed in this study is available from the corresponding author on reasonable request.

## Abbreviations

BMI: Body Mass Index
child-nHDS: Children’s non-Healthy Diet Score
child-pHDS: Children’s pro-Healthy Diet Score
child-UPFs: Children’s Ultra-Processed Food Score
CI: Confidence interval
FFQ: Food Frequency Questionnaire
ICC: Intraclass correlation coefficient
LOA: Limits of agreement
NA: Not applied
PITNUTS: Polish Infant and Toddler Nutrition Study
SD: Standard deviation
UPF: Ultra-processed food
WHO: World Health Organization

## References

[1] Rippin HL, Hutchinson J, Jewell J, Breda JJ, Cade JE. Child and adolescent nutrient intakes from current national dietary surveys of European populations. Nutr Res Rev. 2019;32(1):38–69. 10.1017/S0954422418000161 PMC6536833 30388967

[2] Luque V, Escribano J, Closa-Monasterolo R, Zaragoza-Jordana M, Ferré N, Grote V, et al. Unhealthy dietary patterns established in infancy track to mid-childhood: the EU childhood obesity project. J Nutr. 2018;148(5):752–9. 10.1093/jn/nxy025 29982656

[3] Beluska-Turkan K, Korczak R, Hartell B, Moskal K, Maukonen J, Alexander DE, et al. Nutritional gaps and supplementation in the first 1000 days. Nutrients. 2019;11(12):2891. 10.3390/nu11122891 PMC6949907 31783636

[4] Cade J, Thompson R, Burley V, Warm D. Development, validation and utilisation of food-frequency questionnaires - a review. Public Health Nutr. 2002;5(4):567–87. 10.1079/PHN2001318 12186666

[5] Gibson R. Principles of nutritional assessment. 2nd ed. New York: Oxford University Press; 2005.

[6] Lovell A, Bulloch R, Wall CR, Grant CC. Quality of food-frequency questionnaire validation studies in the dietary assessment of children aged 12 to 36 months: a systematic literature review. J Nutr Sci. 2017;6:e16. 10.1017/jns.2017.12 PMC5468742 28630693

[7] Gonzalez-Palacios S, Ojeda-Belokon C, Oncina-Canovas A, Compañ-Gabucio LM, Pastor-Fajardo MT, Pérez A, et al. Validity of a short food frequency questionnaire for toddlers of nela birth cohort study. Nutrients. 2024;16(24):4403. 10.3390/nu16244403 PMC11677162 39771024

[8] Mason M, Shaw SC, Baird J, Barrett M, Lovelock D, Woods-Townsend K, et al. Development of two short FFQ to assess diet quality in UK pre-school and primary school-aged children based on National Diet and Nutrition Survey data. Br J Nutr. 2025;133(9):1287–96. 10.1017/S0007114525103449 PMC12229983 40383982

[9] Zheng M, Campbell KJ, Scanlan E, McNaughton SA. Development and evaluation of a food frequency questionnaire for use among young children. PLoS ONE. 2020;15(3):e0230669. 10.1371/journal.pone.0230669 PMC7094848 32210467

[10] Wadolowska L, Stasiewicz B. The manual for developing of nutritional data from the KomPAN^®^ questionnaire. Olsztyn: Committee of Human Nutrition, Polish Academy of Sciences; 2024. [Available from: https://diettools4u.uwm.edu.pl/kompan/.

[11] Babio N, de Las Heras-Delgado S, De Miguel-Etayo P, Pastor-Villaescusa B, Leis R, Garcidueñas-Fimbres TE, et al. Reproducibility and relative validity of a semi-quantitative food and beverage frequency questionnaire for Spanish children aged 3 to 11 years: the COME-Kids F&B-FQ. Eur J Pediatr. 2023;182(12):5577–89. 10.1007/s00431-023-05220-9 PMC10746573 37798446

[12] Paladino N, Di Costanzo G, Ruggiero E, Di Castelnuovo A, Dello Russo M, Formisano A, et al. Validity and Reliability of a Food Frequency Questionnaire (NFFQ-Kids) to Assess Food Consumption Based on the Nova Classification in Southern Italian Children and Adolescents. Nutrients. 2025;17(23):3751. 10.3390/nu17233751 PMC12694190 41374041

[13] Livingstone MB, Robson PJ, Wallace JM. Issues in dietary intake assessment of children and adolescents. Br J Nutr. 2004;92(Suppl 2):S213–22. 10.1079/bjn20041169 15522159

[14] Watson EO, Heath AL, Taylor RW, Mills VC, Barris AC, Skidmore PM. Relative validity and reproducibility of an FFQ to determine nutrient intakes of New Zealand toddlers aged 12–24 months. Public Health Nutr. 2015;18(18):3265–71. 10.1017/S1368980015000841 PMC10271836 25824599

[15] Judd AL, Beck KL, McKinlay C, Jackson A, Conlon CA. Validation of a complementary food frequency questionnaire to assess infant nutrient intake. Matern Child Nutr. 2020;16(1):e12879. 10.1111/mcn.12879 PMC7038889 31343830

[16] Vioque J, Gimenez-Monzo D, Navarrete-Muñoz EM, Garcia-de-la-Hera M, Gonzalez-Palacios S, Rebagliato M, et al. Reproducibility and validity of a food frequency questionnaire designed to assess diet in children aged 4–5 years. PLoS ONE. 2016;11(11):e0167338. 10.1371/journal.pone.0167338 PMC5127574 27898731

[17] Weker H, Barańska M, Riahi A, Strucińska M, Więch M, Rowicka G, et al. Nutrition of infants and young children in Poland - Pitnuts 2016. Dev Period Med. 2017;21(1):13–28. 10.34763/devperiodmed.20172101.1328 PMC8522991 28551688

[18] Sawicki M, Kowalkowska J, Kułaga Z, Socha P, Swiąder-Lesniak A, Swiecicka-Ambroziak A, et al. Assessment of dietary practices and nutritional status of children aged 5 to 72 months: study protocol of a nationwide Polish cross-sectional study (PITNUTS 2024). BMJ Open. 2025;15(11):e100921. 10.1136/bmjopen-2025-100921 PMC12636990 41263840

[19] Conway RE, Heuchan GN, Heggie L, Rauber F, Lowry N, Hallen H, et al. Ultra-processed food intake in toddlerhood and mid-childhood in the UK: cross sectional and longitudinal perspectives. Eur J Nutr. 2024;63(8):3149–60. 10.1007/s00394-024-03496-7 PMC11519182 39363048

[20] Chamarthi VS, Shirsat P, Sonavane K, Parsi S, Ravi U, Ponnam HC, et al. The impact of ultra-processed foods on pediatric health. Obes Pillars. 2025;16:100203. 10.1016/j.obpill.2025.100203 PMC12685501 41368198

[21] Kowalkowska J, Wadolowska L, Czarnocinska J, Czlapka-Matyasik M, Galinski G, Jezewska-Zychowicz M, et al. Reproducibility of a questionnaire for dietary habits, lifestyle and nutrition knowledge assessment (KomPAN) in polish adolescents and adults. Nutrients. 2018;10(12):1845. 10.3390/nu10121845 PMC6315932 30513711

[22] Kowalkowska J, Wadolowska L, Hamulka J, Wojtas N, Czlapka-Matyasik M, Kozirok W, et al. Reproducibility of a Short-Form, Multicomponent Dietary Questionnaire to Assess Food Frequency Consumption, Nutrition Knowledge, and Lifestyle (SF-FFQ4PolishChildren) in Polish Children and Adolescents. Nutrients. 2019;11(12):2929. 10.3390/nu11122929 PMC6950380 31816859

[23] Weker H, Friedrich M, Zabłocka-Słowińska K, Sadowska J, Hamułka J, Długosz A, et al. Position statement of the polish academy of sciences’ committee of human nutrition science on the principles for the nutrition of children aged 1–3 years. Standardy Medyczne/Pediatria. 2022;19(4):387–402. 10.32394/rpzh.2022.0238 36546865

[24] Weker H, Friedrich M, Zabłocka-Słowińska K, Sadowska J, Długosz A, Hamułka J. Stanowisko Komitetu Nauki o Żywieniu Człowieka Polskiej Akademii Nauk w sprawie zasad żywienia dzieci w wieku przedszkolnym (4–6 lat) i wczesnoszkolnym (7–9 lat). Standardy Medyczne/Pediatria. 2023;20(4):481–503.

[25] Monteiro C, Cannon G, Lawrence M, Costa Louzada M, Pereira Machado P. Ultra-processed foods, diet quality, and health using the NOVA classification system. Rome: FAO; 2019.

[26] Khandpur N, Rossato S, Drouin-Chartier JP, Du M, Steele EM, Sampson L, et al. Categorising ultra-processed foods in large-scale cohort studies: evidence from the Nurses’ Health Studies, the Health Professionals Follow-up Study, and the Growing Up Today Study. J Nutr Sci. 2021;10:e77. 10.1017/jns.2021.72 PMC8453454 34589209

[27] Kowalkowska J, Wadolowska L, Czarnocinska J, Galinski G, Dlugosz A, Loboda D, et al. Data-driven dietary patterns and diet quality scores: reproducibility and consistency in sex and age subgroups of poles aged 15–65 years. Nutrients. 2020;12(12):3598. 10.3390/nu12123598 PMC7759929 33255188

[28] Masson LF, McNeill G, Tomany JO, Simpson JA, Peace HS, Wei L, et al. Statistical approaches for assessing the relative validity of a food-frequency questionnaire: use of correlation coefficients and the kappa statistic. Public Health Nutr. 2003;6(3):313–21. 10.1079/PHN2002429 12740081

[29] Landis J, Koch G. The measurement of observer agreement for categorical data. Biometrics. 1977;33(1):159–74. 843571

[30] Stanisz A. Przystępny kurs Statystyki z Zastosowaniem STATISTICA PL na Przykładach z Medycyny (Accessible Course of Statistics with Application of STATISTICA PL Using Medical Examples). Kraków: StatSoft; 2006.

[31] Koo TK, Li MY. A guideline of selecting and reporting intraclass correlation coefficients for reliability research. J Chiropr Med. 2016;15(2):155–63. 10.1016/j.jcm.2016.02.012 PMC4913118 27330520

[32] Bland JM, Altman DG. Statistical methods for assessing agreement between two methods of clinical measurement. Lancet. 1986;1(8476):307–10. 2868172

[33] Tavakol M, Dennick R. Making sense of Cronbach’s alpha. Int J Med Educ. 2011;2:53–5. 10.5116/ijme.4dfb.8dfd PMC4205511 28029643

[34] De Onis M. World Health Organization Reference Curves. In: Frelut ML, editor. The ECOG’s eBook on child and adolescent obesity. Brussels: European Childhood Obesity Group; 2015.

[35] Buch-Andersen T, Pérez-Cueto FJ, Toft U. Relative validity and reproducibility of a parent-administered semi-quantitative FFQ for assessing food intake in Danish children aged 3–9 years. Public Health Nutr. 2016;19(7):1184–94. 10.1017/S136898001500275X PMC10271183 26434501

[36] Qin Y, Deng H, Lu M, Cheng Y, Mi B, Xiao Y, et al. Reproducibility and validity of a semi-quantitative food frequency questionnaire for children aged 6–12 in Western China. Nutrients. 2023;15(4):856. 10.3390/nu15040856 PMC9959506 36839215

[37] Lanfer A, Hebestreit A, Ahrens W, Krogh V, Sieri S, Lissner L, et al. Reproducibility of food consumption frequencies derived from the Children’s Eating Habits Questionnaire used in the IDEFICS study. Int J Obes (Lond). 2011;35(Suppl 1):S61–8. 10.1038/ijo.2011.36 21483424

[38] Koleilat M, Whaley SE. Reliability and validity of food frequency questions to assess beverage and food group intakes among low-income 2- to 4-year-old children. J Acad Nutr Diet. 2016;116(6):931–9. 10.1016/j.jand.2016.02.014 27050726

[39] Zec S, Soriani N, Comoretto R, Baldi I. High agreement and high prevalence: the paradox of Cohen’s Kappa. Open Nurs J. 2017;11:211–8. 10.2174/1874434601711010211 PMC5712640 29238424

[40] Huybrechts I, Vereecken C, De Bacquer D, Vandevijvere S, Van Oyen H, Maes L, et al. Reproducibility and validity of a diet quality index for children assessed using a FFQ. Br J Nutr. 2010;104(1):135–44. 10.1017/S0007114510000231 20214836

[41] Lim HX, Toh JY, Tan KH, Chong YS, Yap F, Godfrey KM, et al. Validation of a semi-quantitative FFQ for 18-month-old toddlers: the Growing Up in Singapore Towards Healthy Outcomes (GUSTO) study. Public Health Nutr. 2019;22(11):1990–2000. 10.1017/S1368980019000557 PMC10260654 30940257

[42] Voortman T, Kiefte-de Jong JC, Geelen A, Villamor E, Moll HA, de Jongste JC, et al. The development of a diet quality score for preschool children and its validation and determinants in the Generation R Study. J Nutr. 2015;145(2):306–14. 10.3945/jn.114.199349 25644352

[43] Chen LW, Fung SM, Fok D, Leong LP, Toh JY, Lim HX, et al. The development and evaluation of a diet quality index for Asian toddlers and its perinatal correlates: the GUSTO cohort study. Nutrients. 2019;11(3):535. 10.3390/nu11030535 PMC6472180 30832217

[44] Burrows TL, Collins K, Watson J, Guest M, Boggess MM, Neve M, et al. Validity of the Australian recommended food score as a diet quality index for pre-schoolers. Nutr J. 2014;13:87. 10.1186/1475-2891-13-87 PMC4162955 25178263

[45] Ríos EM, Sinigaglia O, Diaz B, Campos M, Palacios C. Development of a diet quality score for infants and toddlers and its association with weight. J Nutrit Health Food Sci 2016;4(4):10.15226/jnhfs.2016.00171. 10.15226/jnhfs.2016.00171 PMC5283385 28154840

[46] Switkowski KM, Kronsteiner-Gicevic S, Rifas-Shiman SL, Lightdale JR, Oken E. Evaluation of the prime diet quality score from early childhood through mid-adolescence. J Nutr. 2024;154(6):1890–906. 10.1016/j.tjnut.2024.04.014 PMC11217026 38614240

[47] Devenish G, Mukhtar A, Begley A, Do L, Scott J. Development and relative validity of a food frequency questionnaire to assess intakes of total and free sugars in Australian toddlers. Int J Environ Res Public Health. 2017;14(11):1361. 10.3390/ijerph14111361 PMC5708000 29117133

[48] Fangupo LJ, Haszard JJ, Leong C, Heath AM, Fleming EA, Taylor RW. Relative validity and reproducibility of a food frequency questionnaire to assess energy intake from minimally processed and ultra-processed foods in young children. Nutrients. 2019;11(6):1290. 10.3390/nu11061290 PMC6627316 31181631

[49] Chen ZH, Mousavi S, Mandhane PJ, Simons E, Turvey SE, Moraes TJ, et al. Ultraprocessed food consumption and obesity development in Canadian children. JAMA Netw Open. 2025;8(1):e2457341. 10.1001/jamanetworkopen.2024.57341 PMC11786234 39888617

[50] Rosales-Arreola LF, Ayala-Galván C, Villa-Aguilar E, Villegas-González A, Soriano-Rosales RE, Pérez-Guillé B, et al. How to evaluate ultra-processed food intake in pediatrics? UPF score: an easy alternative. BMC Nutr. 2025;11(1):77. 10.1186/s40795-025-01056-8 PMC11998133 40234997

[51] Weker H, Barańska M, Riahi A, Socha P. PITNUTS 2016 study report. Comprehensive evaluation of feeding practices in children aged 5 to 36 months – Polish nationwide study 2016. Warsaw: Institute of Mother and Child; 2021 [Available from: https://fundacjanutricia.pl/uploads/research-files/February2021/Q4zDWNRzLYTa8nZeNasZ.pdf.

[52] McPherson RS, Hoelscher DM, Alexander M, Scanlon KS, Serdula MK. Dietary assessment methods among school-aged children: validity and reliability. Prev Med. 2000;31(2):S11–33.

[53] Palacios C, Rivas-Tumanyan S, Santiago-Rodríguez EJ, Sinigaglia O, Ríos EM, Campos M, et al. A Semi-quantitative food frequency questionnaire validated in hispanic infants and toddlers aged 0 to 24 months. J Acad Nutr Diet. 2017;117(4):526–e359. 10.1016/j.jand.2016.12.010 PMC5382983 28188114

[54] Bjørnarå HB, Hillesund ER, Torstveit MK, Stea TH, Øverby NC, Bere E. An assessment of the test-retest reliability of the New Nordic Diet score. Food Nutr Res. 2015;59:28397. 10.3402/fnr.v59.28397 PMC4534623 26268707

[55] Vereecken C, De Pauw A, Van Cauwenbergh S, Maes L. Development and test-retest reliability of a nutrition knowledge questionnaire for primary-school children. Public Health Nutr. 2012;15(9):1630–8. 10.1017/S1368980012002959 22691728

[56] Sawicki M, Kowalkowska J, Kawiak-Jawor E, Kulaga Z, Rowicka G, Socha P, et al. Diet quality and nutrient adequacy among polish children: findings from the PITNUTS 2024 study. Nutrients. 2025;17:21. 10.3390/nu17213364 PMC12611091 41228438

[57] Sochacka-Tatara E, Pac A. Relative validity of a semi-quantitative FFQ in 3-year-old Polish children. Public Health Nutr. 2014;17(8):1738–44. 10.1017/S1368980013002292 PMC10282492 24172011

[58] Wingrove K, Lawrence MA, McNaughton SA. A systematic review of the methods used to assess and report dietary patterns. Front Nutr. 2022;9:892351. 10.3389/fnut.2022.892351 PMC9175067 35694166

[59] Krusinska B, Hawrysz I, Wadolowska L, Slowinska MA, Biernacki M, Czerwinska A, et al. Associations of mediterranean diet and a posteriori derived dietary patterns with breast and lung cancer risk: a case-control study. Nutrients. 2018;10(4):470. 10.3390/nu10040470 PMC5946255 29641468

[60] Trichopoulou A, Costacou T, Bamia C, Trichopoulos D. Adherence to a Mediterranean diet and survival in a Greek population. N Engl J Med. 2003;348(26):2599–608. 10.1056/NEJMoa025039 12826634

