# Supplementary material for: Reproducibility of an 85-item Child Food Frequency Questionnaire (85-item Child-FFQ) and diet quality scores among Polish children: The cross-sectional PITNUTS 2024 study
Source: BMC Pediatr. 2026 Jul 8;26:949. doi: 10.1186/s12887-026-07222-6 (PMC13640158; doi:10.1186/s12887-026-07222-6)
Supplement: Supplementary file 1 [file 12887_2026_7222_MOESM1_ESM.docx]

# Reproducibility of an 85-item Child Food Frequency Questionnaire (85-item Child-FFQ) and diet quality scores among Polish children: The cross-sectional PITNUTS 2024 study

**Supplementary materials**

Table S1. Components of the Children's pro-Healthy Diet Score developed for children aged 13-72 months (child-pHDS)

| No. | Components  of the child-pHDS | No. of  FFQ item | Items of the FFQ |
| --- | --- | --- | --- |
| 1 | Vegetables | 37 | Vegetables (fresh/frozen) |
|  |  | 38 | Vegetables (e.g. boiled, including soups) |
|  |  | 40 | Pickled vegetables |
| 2 | Pulses/nuts | 41 | Pulses and pulse-based dishes |
|  |  | 49 | Nuts, seeds |
| 3 | Fruit | 45 | Fruit (fresh/frozen) |
|  |  | 46 | Fruit mousse |
| 4 | Cereal products | 2 | Wholemeal bread |
|  |  | 5 | Brown rice |
|  |  | 7 | Whole grain pasta |
|  |  | 8 | Groats |
|  |  | 11 | Natural cereals (e.g. oat flakes) |
| 5 | Unsweetened dairy products | 15 | Whole milk |
|  |  | 16 | Semi-skimmed milk |
|  |  | 17 | Skimmed milk |
|  |  | 21 | Natural yogurt, kefir, buttermilk |
|  |  | 23 | Cottage cheese and other unsweetened cheeses |
| 6 | Meat/fish/eggs | 28 | White meat |
|  |  | 29 | Red meat |
|  |  | 32 | Fresh/frozen fish |
|  |  | 35 | Seafood |
|  |  | 36 | Eggs |
| 7 | Recommended fats | 51 | Butter |
|  |  | 54 | Olive oil |
|  |  | 55 | Vegetable oils |

Table S2. Components of the Children's non-Healthy Diet Score developed for children aged 13-72 months (child-nHDS)

| No. | Components  of the child-nHDS | No. of  FFQ item | Items of the FFQ |
| --- | --- | --- | --- |
| 1 | Sweetened breakfast cereals | 9 | Flavoured breakfast cereals |
| 2 | Fried flour-based dishes | 13 | Fried flour dishes (e.g. pancakes) |
| 3 | Fast food | 14 | Fast food (excluding French fries) |
|  |  | 43 | French fries |
| 4 | Sweetened dairy products | 18 | Flavoured milk beverages (e.g. cocoa) |
|  |  | 19 | Flavoured condensed milk |
|  |  | 22 | Flavoured yogurt, buttermilk |
|  |  | 24 | Flavoured homogenized cheeses |
|  |  | 25 | Dairy desserts (e.g. pudding) |
| 5 | Processed meat | 30 | High-quality cold cuts |
|  |  | 31 | Sausages, pates |
| 6 | Food concentrates/ salty seasonings | 59 | Salty condiments with vegetables (e.g. bouillon cubes) |
|  |  | 60 | Instant soups and sauces |
| 7 | Sweet condiments | 61 | Sugar |
|  |  | 63 | Fruit syrups |
|  |  | 64 | Jam, marmalade |
|  |  | 65 | Chocolate/chocolate-nut spreads |
| 8 | Sweets | 66 | Chocolate, chocolate bars |
|  |  | 67 | Candies |
|  |  | 68 | Sponge cookies, biscuits |
|  |  | 69 | Other cookies |
|  |  | 70 | Cakes and pastries |
| 9 | Salty snacks | 72 | Salty snacks |
| 10 | Sweetened beverages | 74 | Flavoured water (commercial) |
|  |  | 75 | Sweetened tea |
|  |  | 77 | Fruit compotes (sweetened) |
|  |  | 84 | Sugar-sweetened soft drinks |
|  |  | 85 | Diet soft drinks |

Table S3. Components of the Children's Ultra-Processed Food Score developed for children aged 13-72 months (child-UPFs)

| No. | Components  of the child-UPFs | No. of  FFQ item | Items of the FFQ |
| --- | --- | --- | --- |
| 1 | Ready-to-eat breakfast cereals | 9 | Flavoured breakfast cereals |
|  |  | 10 | Corn flakes (unsweetened) |
| 2 | Fast food | 14 | Fast food (excluding French fries) |
|  |  | 43 | French fries |
| 3 | Sweetened dairy products | 18 | Flavoured milk beverages (e.g. cocoa) |
|  |  | 19 | Flavoured condensed milk |
|  |  | 22 | Flavoured yogurt, buttermilk |
|  |  | 24 | Flavoured homogenized cheeses |
|  |  | 25 | Dairy desserts (e.g. pudding) |
| 4 | Processed meat | 30 | High-quality cold cuts |
|  |  | 31 | Sausages, pates |
| 5 | Savoury condiments | 44 | Ketchup |
|  |  | 53 | Margarines, butter and margarine mixes |
|  |  | 56 | Mayonnaise |
| 6 | Food concentrates/ salty seasonings | 59 | Salty condiments with vegetables  (e.g. bouillon cubes) |
|  |  | 60 | Instant soups and sauces |
| 7 | Sweet condiments | 63 | Fruit syrups |
|  |  | 65 | Chocolate/chocolate-nut spreads |
| 8 | Sweets | 66 | Chocolate, chocolate bars |
|  |  | 67 | Candies |
|  |  | 68 | Sponge cookies, biscuits |
|  |  | 69 | Other cookies |
| 9 | Salty snacks | 72 | Salty snacks |
| 10 | Beverages | 74 | Flavoured water (commercial) |
|  |  | 82 | Plant-based beverages |
|  |  | 84 | Sugar-sweetened soft drinks |
|  |  | 85 | Diet soft drinks |

Table S4. Median daily consumption frequency [times/day] of food groups constituting the components of diet quality scores developed for children aged 13-72 months

| Median consumption frequency [times/day] | Children’s age groups | |
| --- | --- | --- |
|  | 13–36 months | 37–72 months |
| Components of the child-pHDS |  |  |
| Vegetables | 1.28 | 1.28 |
| Pulses/nuts | 0.06 | 0.12 |
| Fruit | 1.06 | 1.00 |
| Cereal products | 0.60 | 0.77 |
| Unsweetened dairy products | 0.69 | 1.06 |
| Meat/fish/eggs | 1.28 | 1.34 |
| Recommended fats | 1.14 | 1.67 |
| Components of the child-nHDS |  |  |
| Sweetened breakfast cereals | 0.06 | 0.14 |
| Fried flour-based dishes | 0.14 | 0.14 |
| Fast food | 0.06 | 0.12 |
| Sweetened dairy products | 0.63 | 0.85 |
| Processed meat | 0.71 | 1.14 |
| Food concentrates/salty seasonings | 0.06 | 0.14 |
| Sweet condiments | 0.42 | 0.91 |
| Sweets | 0.57 | 0.83 |
| Salty snacks | 0.06 | 0.06 |
| Sweetened beverages | 0.34 | 0.79 |
| Components of the child-UPFs |  |  |
| Ready-to-eat breakfast cereals | 0.14 | 0.57 |
| Fast food | 0.06 | 0.12 |
| Sweetened dairy products | 0.63 | 0.85 |
| Processed meat | 0.71 | 1.14 |
| Savoury condiments | 0.06 | 0.26 |
| Food concentrates/salty seasonings | 0.06 | 0.14 |
| Sweet condiments | 0.06 | 0.12 |
| Sweets | 0.56 | 0.57 |
| Salty snacks | 0.06 | 0.06 |
| Beverages | 0.00 | 0.12 |

Table S5. Internal reliability of the Children's pro-Healthy Diet Score developed for children aged 13-72 months (child-pHDS) – Cronbach’s alpha values for the child-pHDS and after excluding a given component

| No. | Components of the child-pHDS | Children’s age groups | |
| --- | --- | --- | --- |
|  |  | 13-36 months | 37-72 months |
|  | Sample size | 400 | 300 |
|  | Child-pHDS | 0.678 | 0.782 |
| 1 | Vegetables | 0.613 | 0.739 |
| 2 | Pulses/nuts | 0.661 | 0.759 |
| 3 | Fruit | 0.673 | 0.766 |
| 4 | Cereal products | 0.640 | 0.770 |
| 5 | Unsweetened dairy products | 0.643 | 0.724 |
| 6 | Meat/fish/eggs | 0.614 | 0.733 |
| 7 | Recommended fats | 0.652 | 0.780 |

Table S6. Internal reliability of the Children's non-Healthy Diet Score developed for children aged 13-72 months (child-nHDS) – Cronbach’s alpha values for the child-nHDS and after excluding a given component

| No. | Components of the child-nHDS | Children’s age groups | |
| --- | --- | --- | --- |
|  |  | 13-36 months | 37-72 months |
|  | Sample size | 400 | 300 |
|  | Child-nHDS | 0.783 | 0.766 |
| 1 | Sweetened breakfast cereals | 0.776 | 0.760 |
| 2 | Fried flour-based dishes | 0.777 | 0.752 |
| 3 | Fast food | 0.776 | 0.756 |
| 4 | Sweetened dairy products | 0.751 | 0.747 |
| 5 | Processed meat | 0.757 | 0.746 |
| 6 | Food concentrates/salty seasonings | 0.774 | 0.753 |
| 7 | Sweet condiments | 0.730 | 0.714 |
| 8 | Sweets | 0.746 | 0.721 |
| 9 | Salty snacks | 0.778 | 0.755 |
| 10 | Sweetened beverages | 0.762 | 0.746 |

Table S7. Internal reliability of the Children's Ultra-Processed Food Score developed for children aged 13-72 months (child-UPFs) – Cronbach’s alpha values for the child-UPFs and after excluding a given component

| No. | Components of the child-UPFs | Children’s age groups | |
| --- | --- | --- | --- |
|  |  | 13-36 months | 37-72 months |
|  | Sample size | 400 | 300 |
|  | Child-UPFs | 0.770 | 0.772 |
| 1 | Ready-to-eat breakfast cereals | 0.756 | 0.752 |
| 2 | Fast food | 0.753 | 0.750 |
| 3 | Sweetened dairy products | 0.733 | 0.757 |
| 4 | Processed meat | 0.754 | 0.759 |
| 5 | Savoury condiments | 0.749 | 0.741 |
| 6 | Food concentrates/salty seasonings | 0.758 | 0.761 |
| 7 | Sweet condiments | 0.742 | 0.747 |
| 8 | Sweets | 0.738 | 0.742 |
| 9 | Salty snacks | 0.762 | 0.761 |
| 10 | Beverages | 0.758 | 0.757 |
